# Anatomically refined entorhinal cortex segmentation improves MRI-based early diagnosis of Alzheimer’s disease

**DOI:** 10.3389/fnagi.2025.1682106

**Published:** 2025-12-03

**Authors:** Yongha Gi, Sangyoon Park, Hyungjin Lim, Jeongwon Lee, A. Hyun Jung, Seol-Hee Baek, Jong Hyun Kim, Byung-Jo Kim, Myonggeun Yoon

**Affiliations:** 1 Department of Biomedical Engineering, Korea University, Seoul, Republic of Korea; 2 FieldCure Co., Ltd., Seoul, Republic of Korea; 3 College of Medicine, Korea University, Seoul, Republic of Korea

**Keywords:** Alzheimer’s disease, entorhinal cortex segmentation, neuroimaging biomarkers, perirhinal cortex, structural MRI

## Abstract

**Introduction:**

The entorhinal cortex (EC) is one of the earliest cortical regions affected in Alzheimer’s disease (AD) and serves as a key target for magnetic resonance imaging (MRI) biomarkers. However, conventional segmentation pipelines based on the Desikan–Killiany atlas do not clearly distinguish the EC from the adjacent perirhinal cortex, leading to mixed labels and reduced diagnostic sensitivity.

**Methods:**

To address these anatomical ambiguities, we developed a refined EC segmentation framework that combines expert-guided anatomical correction with deep learning. FreeSurfer-derived EC labels were manually refined by removing anterior perirhinal extensions and other anatomically inconsistent regions that are functionally distinct from the EC. These expert-corrected labels were then used to train a no-new-Net (nnU-Net) model on Alzheimer’s Disease Neuroimaging Initiative 1 (ADNI1) MRI data, enabling anatomically precise and scalable EC delineation across individuals and scanners.

**Results:**

The refined EC segmentation preserved anatomically valid boundaries and demonstrated stronger group-level differentiation among cognitively normal, mild cognitive impairment, and AD groups. When incorporated into volumetric and classification analyses, it provided more specific imaging biomarkers of early neurodegeneration and improved discrimination between diagnostic stages. External validation further confirmed reliable generalization across datasets.

**Discussion:**

These findings demonstrate that anatomically precise and expert-informed EC delineation improves the sensitivity of MRI-based biomarkers for early AD diagnosis. The proposed framework offers a practical and reproducible approach for studying subtle cortical changes that precede overt clinical symptoms.

## 1 Introduction

Alzheimer’s disease (AD) is the most prevalent neurodegenerative disorder, affecting over 55 million individuals globally and accounting for approximately 60–80% of all dementia cases (Alzheimer’s Disease International. World Alzheimer Report, 2021). As the disease progresses, patients experience a gradual and irreversible decline in cognitive function, ultimately resulting in a loss of independence. In addition to its devastating personal toll, AD poses a significant socioeconomic burden, particularly as global life expectancy increases and aging populations continue to grow (Alzheimer’s Disease International. World Alzheimer Report, 2021). In the absence of curative treatments, early and accurate diagnosis is critical for enabling timely therapeutic interventions and improving long-term clinical outcomes. Early-stage detection also facilitates better planning for patients and caregivers and provides an opportunity to enroll individuals in clinical trials targeting prodromal disease phases.

Structural MRI has become a cornerstone modality in AD research due to its noninvasive nature and ability to capture fine-grained anatomical detail. Among MRI-derived biomarkers (Tae et al., 2025), volumetric measures of the hippocampus and other medial temporal structures have long been used to quantify neurodegeneration and support early disease detection (Brewer et al., 2013; Eskildsen et al., 2015). Although hippocampal atrophy remains a well-established imaging marker of AD progression, accumulating evidence indicates that neurodegenerative changes emerge even earlier in the EC (Kulason et al., 2020; Braak and Braak, 1991). Situated in the medial temporal lobe, the EC serves as a key interface between the hippocampus and neocortex, supporting memory consolidation and retrieval. Importantly, numerous studies have demonstrated that EC volume loss closely tracks cognitive decline and often precedes the onset of clinical symptoms, underscoring its potential as a sensitive biomarker for preclinical AD (Eskildsen et al., 2015; Sierra-Marcos, 2017; Khan et al., 2014).

Despite this relevance, reliably delineating the EC from structural MRI remains challenging (Hasan et al., 2016). Conventional atlas-based frameworks such as the Desikan–Killiany (DK) and Desikan–Killiany–Tourville atlases (Alexander et al., 2019), as implemented in FreeSurfer (Fischl, 2012) and FastSurfer (Henschel et al., 2020), define the EC as a single composite label that does not separate it from the adjacent perirhinal cortex. Anatomically, the EC is a thin and highly folded cortical ribbon that borders the perirhinal cortex—part of Brodmann area 35 (BA35)—which is involved in object and semantic memory rather than episodic processing (Augustinack et al., 2013). This boundary ambiguity leads to partial inclusion of perirhinal tissue in EC segmentations, introducing functional heterogeneity that can obscure AD-related atrophy patterns and weaken the diagnostic specificity of EC-based biomarkers (Augustinack et al., 2013; Fung et al., 2017).

Previous probabilistic and surface-based atlases have improved intersubject alignment but still fail to reflect the cytoarchitectonic distinction of BA35 (Kulason et al., 2020; Fung et al., 2017). Consequently, existing EC segmentations often conflate structurally adjacent but functionally distinct cortical regions. Functional MRI and histological studies have consistently demonstrated that the entorhinal and perirhinal cortices exhibit distinct connectivity, activation, and pathological trajectories: tau pathology typically emerges in the transentorhinal (perirhinal) region before spreading into the EC proper, while amyloid deposition follows a distinct pattern (Maass et al., 2015). These findings highlight the importance of anatomically refined EC delineation strategies that consider both structural and functional boundaries.

To address these limitations, we developed an anatomically refined EC segmentation strategy that delineates its boundaries more precisely while excluding the adjacent perirhinal cortex, whose partial inclusion in conventional atlases may obscure disease-related atrophy. The refinement was based on histologically defined anatomical boundaries and prior functional findings differentiating the EC from neighboring regions, and was implemented through expert-corrected training labels for a deep learning segmentation model. This study aimed to evaluate whether such anatomically and functionally informed refinement improves the diagnostic performance of MRI-based biomarkers for AD, particularly in distinguishing cognitively normal (CN) individuals, patients with mild cognitive impairment (MCI), and those with AD.

## 2 Materials and methods

### 2.1 Dataset

This study employed structural MRI data from two publicly available sources: the Alzheimer’s Disease Neuroimaging Initiative 1 (ADNI1) (Mueller et al., 2005) and the Minimal Interval Resonance Imaging in Alzheimer’s Disease (MIRIAD) dataset (Malone et al., 2013). These datasets together provided a broad spectrum of clinical stages, ranging from CN individuals to patients with MCI and AD.

ADNI1 served as the primary dataset for both model training and evaluation, offering a large and balanced cohort across diagnostic groups. In contrast, MIRIAD was used exclusively for testing, allowing us to assess the generalizability of our segmentation approach on an external dataset acquired under different imaging conditions. All structural T1-weighted MRI scans were obtained at a standardized isotropic resolution of 1 mm^3^ and preprocessed to a uniform volume matrix of 256 × 256 × 256 voxels. A detailed breakdown of subject composition is summarized in Table 1.

**Table 1:** Dataset composition.

Dataset	Subjects	Women, *n*(%)	CN, *n*(%)	MCI, *n*(%)	AD, *n*(%)
ADNI1	1,423	589 (41)	448 (31)	680 (48)	295 (21)
MIRIAD	69	38 (55)	23 (33)	0 (0)	46 (67)

Summary of the dataset composition used in this study. The ADNI1 and MIRIAD cohorts include a total of 1,423 and 69 subjects, respectively. For each dataset, the number and percentage of female participants, as well as the diagnostic group distributions—CN, MCI, and AD—are provided. No MCI subjects are included in the MIRIAD dataset.

### 2.2 Segmentation approaches

**Figure 1:**
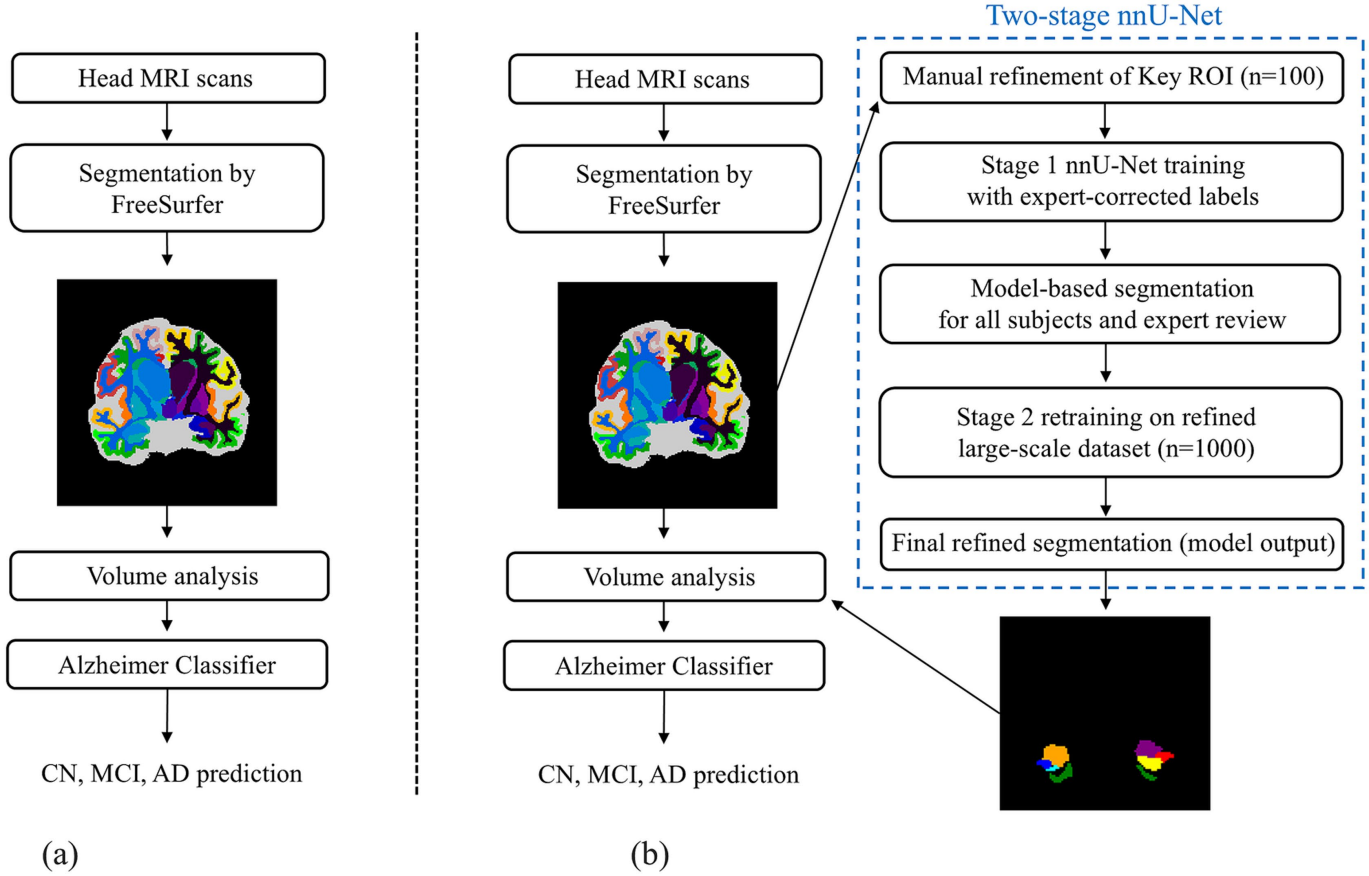
Overview of the two segmentation pipelines for extracting AD–related volumetric biomarkers from structural MRI. **(a)** Conventional segmentation pipeline using FreeSurfer. **(b)** Refined segmentation pipeline employing a two-stage nnU-Net framework trained on expert-corrected labels. For both pipelines, the resulting volumetric features were used for subsequent volume analysis and classification to support diagnosis and disease stage prediction.

We applied two distinct segmentation-driven analysis pipelines to structural MRI scans to extract and utilize volumetric biomarkers relevant to AD (see Figure 1). These pipelines are referred to as Case 1 (Conventional Segmentation) and Case 2 (Refined Segmentation).

Both pipelines begin with brain segmentation following FreeSurfer conventions, which parcellate the cerebral cortex into 68 cortical regions using the DK atlas and divide the entire brain into approximately 114 anatomical structures, including subcortical nuclei, ventricles, white matter, and cerebrospinal fluid compartments. From these segmentations, 8 key AD-relevant brain regions were selected for downstream volumetric analysis, including the hippocampus, amygdala, inferior temporal cortex, inferior lateral ventricle, and most critically, the EC. These extracted volumes served as input features for subsequent machine learning–based classification models aimed at distinguishing diagnostic groups and assessing disease severity.

#### 2.2.1 Case 1: conventional segmentation

In this baseline approach, whole-brain segmentation was performed using FreeSurfer (version 7.4.1) on 1,423 subjects from the ADNI1 dataset. Regional volumes were extracted directly from the standard DK parcellation, as illustrated in Figure 1a. For analytic consistency and to reduce hemispheric noise, left and right EC volumes were combined into a single bilateral measure. Importantly, this method does not distinguish the EC from adjacent perirhinal cortex, as the DK atlas defines the entire region as a single anatomical label.

#### 2.2.2 Case 2: refined EC segmentation

A two-stage no-new-Net (nnU-Net) (Isensee et al., 2021)–based segmentation framework was developed to generate anatomically refined EC labels, as shown in Figure 1b. Manual delineations were created and reviewed by two neurologists from a university-affiliated hospital to serve as the foundation for model training and evaluation.

The correction process was grounded in anatomical landmarks, primarily targeting known sources of over-segmentation in FreeSurfer-based EC labels. Specifically, mislabeled voxels corresponding to anterior perirhinal cortex (BA 35), adjacent meninges, and lateral extensions into the collateral sulcus were carefully removed. The refined EC label was constrained to the medial bank of the collateral sulcus and posterior entorhinal extent, following established anatomical boundaries from high-resolution histological and structural MRI studies. In addition, previously published functional connectivity maps were qualitatively consulted to guide the anterior limit of the EC and to support exclusion of perirhinal regions known to be functionally distinct. This adaptive two-stage refinement follows an iterative design in which model retraining progressively improves convergence stability and boundary accuracy (Ali et al., 2024).

In the first stage, nnU-Net was trained on 100 manually corrected subjects from the ADNI1 dataset (34 CN, 33 MCI, and 33 AD). The trained model was then applied to the entire ADNI1 cohort (*n* = 1,423) to generate initial model-derived EC segmentations. To ensure anatomical fidelity, all model-generated labels were subsequently reviewed and manually corrected by the same neurologists using a structured post-processing protocol conceptually similar to workflows in large-scale efforts such as TotalSegmentator (Wasserthal et al., 2023), where automated outputs are systematically verified by human experts.

In the second stage, based on this fully curated set of 1,423 expert-corrected labels, the dataset was randomly divided into 1,000 subjects for training and 423 for independent testing. A second nnU-Net model was retrained on the training subset, and its performance was evaluated against the expert-corrected labels of the held-out test subjects using the Dice Similarity Coefficient (DSC) (Popovic et al., 2007), Surface Dice Similarity Coefficient (SDSC) (Nikolov et al., 2018), 95th-percentile Hausdorff Distance (HD95) (Popovic et al., 2007), and Average Surface Distance (ASD) (Popovic et al., 2007).

### 2.3 Comparative analysis of segmentation methods

To comprehensively evaluate the diagnostic relevance of different EC segmentation strategies, we conducted a three-step comparative analysis comprising group-level statistics, feature importance estimation, and model-based interpretability analyses.

First, group-level statistical comparisons were performed using one-way analysis of variance (ANOVA) (Coolican, 2017) across CN, MCI, and AD cohorts to assess whether each EC segmentation exhibited significant diagnostic effects.

Second, to quantify the diagnostic informativeness of each EC feature, we applied two complementary importance analyses based on Random Forest (RF) (Coolican, 2017) models: (1) Permutation importance, which measured the reduction in classification accuracy when each EC feature was randomly shuffled, and (2) Regression-based importance, which estimated feature relevance by the reduction in mean squared error (MSE) when predicting a continuous disease severity score (CN = 0.0, MCI = 0.5, AD = 1.0). Both analyses were restricted to the two EC features (conventional and refined) to enable a direct, pairwise comparison under identical modeling conditions.

Finally, to interpret the EC’s contribution within a broader anatomical context, we employed SHapley Additive exPlanations (SHAP) (Lundberg and Lee, 2017) using XGBoost (XG) (Chen and Guestrin, 2016) models. Specifically, XG classifiers and regressors were trained on nine volumetric features—eight canonical AD-related regions of interest (ROIs) in which the EC was originally included, but divided into two variants (conventional and refined) for direct comparison. SHAP values were then computed to rank the relative importance of each ROI, allowing a side-by-side evaluation of the conventional and refined EC alongside other major AD-related structures.

This multi-level analysis provided complementary insights into how anatomically refined segmentation enhances the diagnostic specificity of EC volume beyond global atrophy patterns.

### 2.4 Machine learning framework for diagnostic classification

To evaluate the diagnostic utility of the refined segmentations, we classified subjects into three groups: CN, MCI, and AD. The entorhinal volumes from each segmentation case were combined with other key ROI volumes and used to train the classification models. The segmentation-derived volumetric features from each dataset were used as input for five classification models: RF (Breiman, 2001), Logistic Regression (LR) (Cox, 1958), Support Vector Machine (SVM) (Cortes and Vapnik, 1995), XG (Chen and Guestrin, 2016), and Multi-Layer Perceptron (MLP) (Rumelhart et al., 1986). Model performance was evaluated using the F1 score (Rumelhart et al., 1986) and the Area Under the Receiver Operating Characteristic Curve (AUROC) (Powers, 2020), which was selected as the primary metric because it provides a robust measure of classification performance across different class distributions. To assess the statistical significance of performance differences between the conventional and refined segmentations, we conducted paired t-tests on F1-scores across five cross-validation folds and pairwise DeLong tests on AUROC values derived from aggregated out-of-fold (OOF) predictions.

### 2.5 Implementation details

For the segmentation described in Section 2.2, the nnU-Net model (Isensee et al., 2021) was trained using the standard 3D full-resolution configuration. Specifically, we employed stochastic gradient descent with Nesterov momentum (0.99), an initial learning rate of 0.01 with polynomial decay, and a weight decay of 3 × 10^−5^. The loss function combined soft Dice and cross-entropy terms with deep supervision. Patch size and batch size were automatically configured by the nnU-Net planner according to GPU memory. To preserve hemispheric asymmetry and ensure consistent anatomical alignment across subjects and scanners, mirror augmentation was disabled, and all input MR images were aligned according to their affine matrices prior to preprocessing. This configuration stabilized the spatial correspondence of medial temporal structures and yielded improved segmentation performance compared to the default augmentation settings. Training was performed for 1,000 epochs using 3D full-resolution models on the ADNI1 dataset.

For the feature-importance estimation described in Section 2.3, RF classifiers and regressors were implemented. Permutation importance was obtained by randomly shuffling each EC feature and measuring the change in classification accuracy, repeated 500 times and averaged for stability. Regression-based importance was calculated based on the reduction in mean squared error when predicting continuous disease severity, also averaged over 500 iterations. For broader interpretability, XG models were trained and SHAP values were computed using TreeExplainer (SHAP v0.41.0, tree_method = “hist”). Both binary (CN vs. AD) and regression models were evaluated, and SHAP values were computed for 100 held-out samples.

For the classification analyses described in Section 2.4, all volumetric features were normalized using robust z-score normalization (based on median and interquartile range) after outlier clipping (±3 SD). Classification models were trained and evaluated using stratified 5-fold cross-validation. For each classification task (CN vs. AD, CN vs. MCI, MCI vs. AD), five models were trained: RF, LR, SVM, XG, and MLP. The MLP was trained for 1,000 epochs using the AdamW optimizer (learning rate = 1e-5, batch size = 64). A ReduceLROnPlateau scheduler was applied with a factor of 0.5, patience of 5, and minimum learning rate of 1e-13 to prevent overfitting.

All experiments were implemented in Python (v3.8.19) using Scikit-learn (v1.2.2), XGBoost (v1.7.5), PyTorch (v2.0.1), and the SHAP package. Computations were performed on an NVIDIA RTX 4090 GPU.

### 2.6 Ethical considerations

This study utilized only de-identified, publicly available MRI datasets from the ADNI1 and the MIRIAD study. All participants originally provided written informed consent, and data collection procedures were approved by the relevant institutional review boards. The current analysis involved no direct interaction with participants and required no additional ethical approval.

## 3 Results

### 3.1 Comparison of segmentation results

**Figure 2:**
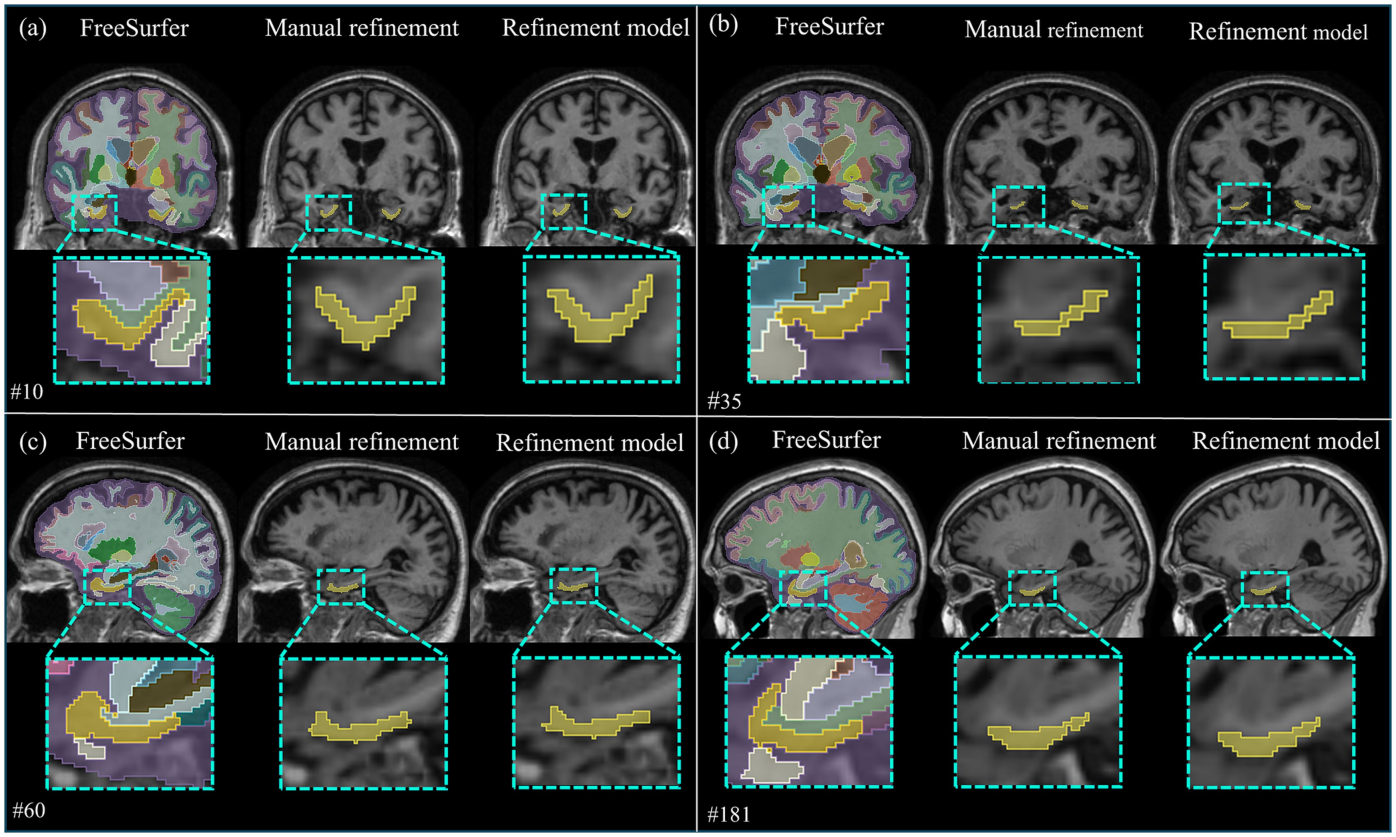
Examples presenting qualitative segmentation results for CN and AD subjects. Panels **(a,c)** show coronal and sagittal planes, respectively, from CN subjects (cases #10 and #60), while panels **(b,d)** display corresponding views from AD subjects (cases #35 and #181).

Figure 2 presents qualitative segmentation outcomes comparing conventional FreeSurfer-based labels with expert-refined labels and the outputs of the second-stage nnU-Net model from our two-stage segmentation framework. Visual comparison reveals that both the expert-annotated and model-generated segmentations exhibit a truncated anterior extent of the EC, consistent with histologically defined posterior boundaries of the EC. In contrast, the FreeSurfer-based segmentations include broader anterior regions, often extending into areas corresponding to the perirhinal cortex. The high visual similarity between the manual and nnU-Net segmentations confirms the model’s ability to reproduce expert-level labeling.

Quantitatively, the retrained second-stage nnU-Net model was evaluated on a held-out test subset (n = 423) that had undergone expert correction. The model achieved a DSC of 0.750, a SDSC of 0.815, a HD95 of 2.50 mm, and an ASD of 0.618 mm. As summarized in Table 2, DSC and SDSC remained stable across diagnostic stages (CN, MCI, and AD), demonstrating consistent volumetric overlap despite progressive cortical deformation. A modest reduction in DSC was observed in the MCI group (0.716), reflecting intermediate morphological variability. HD95 and ASD were lowest in the AD group (1.83 mm and 0.498 mm), suggesting tighter surface correspondence in subjects with advanced atrophy. This observation may partly result from reduced cortical surface area in advanced stages, which can artifactually lower distance-based metrics. Overall, these findings indicate that the refined segmentation framework achieved stable convergence in segmentation accuracy across disease stages. This progressive stabilization process conceptually resembles bio-inspired optimization-based identification strategies (Ali et al., 2025b), in which iterative feedback mechanisms enhance convergence stability and learning efficiency.

**Table 2:** Segmentation performance of the refined EC model across disease stages.

Diagnostic group	DSC	SDSC	HD95	ASD
CN	**0.778**	0.829	2.41	0.655
MCI	0.716	0.784	2.96	0.627
AD	0.755	**0.848**	**1.83**	**0.498**
Mean	0.750	0.815	2.50	0.618

DSC, SDSC, HD95, and ASD are reported for CN, MCI, and AD groups, with the bottom row representing the overall mean across all subjects. The refined model maintained stable overall accuracy and boundary precision across stages, with a moderate DSC reduction in the MCI group, reflecting transitional morphological variability during disease progression. Bold values indicate the best values within each evaluation metric.

**Figure 3:**
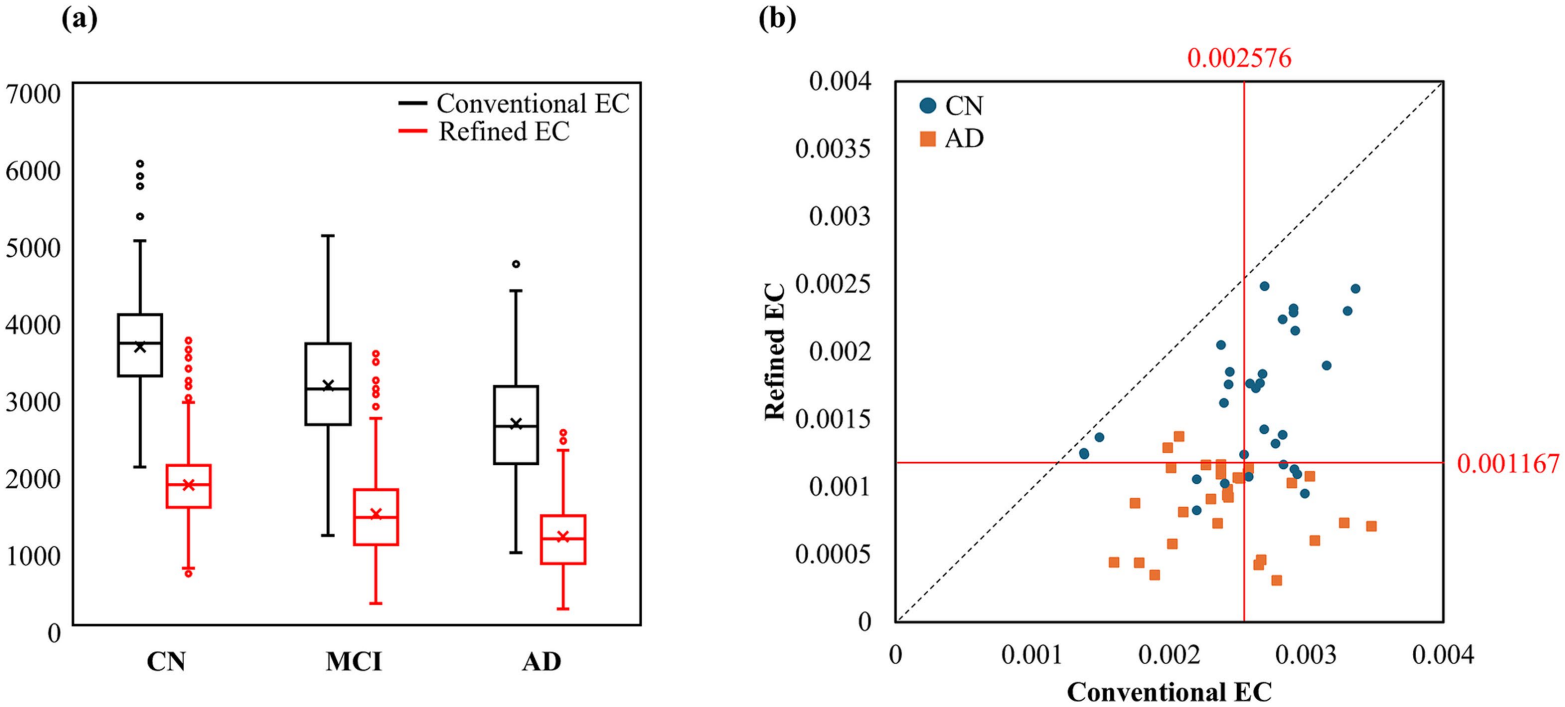
Comparison of conventional and refined EC volumes. **(a)** Absolute EC volumes across CN, MCI, and AD groups. **(b)** ICV-normalized EC volumes in a manually labeled training set, illustrating quadrant-based diagnostic alignment. Red lines indicate the optimal cutoffs determined by maximizing Youden’s J statistic.

Figure 3 further examines volumetric and diagnostic differences between segmentation methods. Figure 3a presents boxplots of absolute EC volumes derived from both conventional and refined segmentations, stratified by diagnostic group (CN, MCI, AD) using the full ADNI1 cohort (*n* = 1,423). For the conventional segmentation, the mean EC volumes were 3,702 mm^3^ (CN), 3,203 mm^3^ (MCI), and 2,708 mm^3^ (AD). For the refined segmentation, the corresponding means were 1,912 mm^3^ (CN), 1,533 mm^3^ (MCI), and 1,240 mm^3^ (AD). This corresponds to proportional reductions to 51.6, 47.9, and 45.8% of the conventional EC volumes for CN, MCI, and AD, respectively. The degree of reduction increased with disease severity, suggesting that the refined approach may more accurately capture AD-related EC atrophy.

Figure 3b presents EC-to-intracranial-volume (ICV) ratios—that is, EC volumes normalized by ICV to account for individual differences in brain size—for a subset of 67 subjects (34 CN and 33 AD) drawn from the initial 100 manually corrected cases used to train the Stage 1 nnU-Net of the two-stage refinement framework. The scatter plot compares conventional (*x*-axis) and refined (*y*-axis) segmentations. The red diagonal line represents the identity line, while the horizontal and vertical lines correspond to thresholds maximizing Youden’s *J* statistic for each method. Notably, in the second quadrant—where the refined EC classified a subject as CN but the conventional EC classified the same subject as AD—there were 8 CN and 3 AD cases. In the fourth quadrant—refined EC indicated AD while conventional EC indicated CN—there were 3 CN and 17 AD cases. This quadrant-based analysis suggests that the refined EC better aligned with true diagnoses. Although only CN and AD groups are visualized in Figure 3b for clarity, *R*^2^ values were computed for all three diagnostic groups (CN = 0.41, MCI = 0.046, AD ≈ 0), further highlighting the distinct nature of the two segmentation methods.

**Figure 4:**
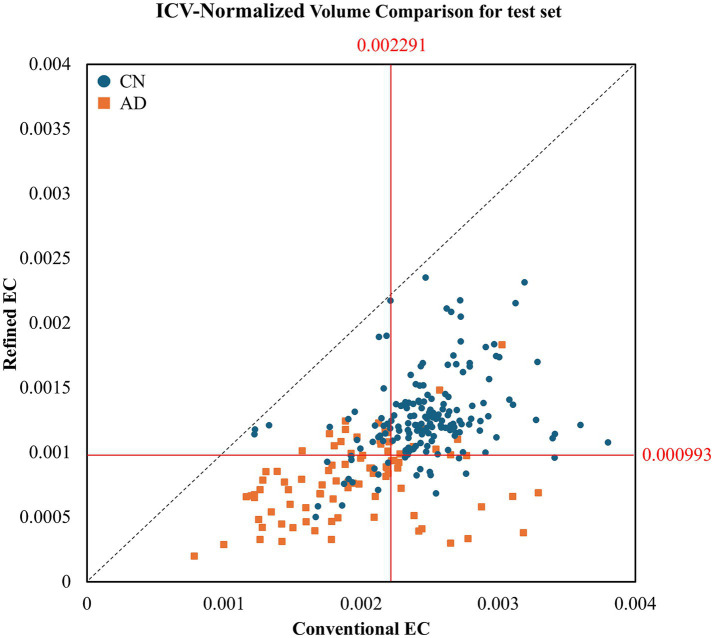
ICV-normalized EC volumes in an independent test set, illustrating quadrant-based diagnostic alignment. Red lines indicate the optimal cutoffs determined by maximizing Youden’s J statistic on the test set.

Figure 4 presents a similar analysis using data from a separate, independent test subset of 260 subjects (CN + AD), drawn from the 423 cases used to evaluate the Stage 2 (retrained) nnU-Net of the two-stage refinement framework. This test set was held out during retraining and used to assess the generalization performance of the final model. Here, the quadrant-based pattern persisted. In the second quadrant (refined: CN, conventional: AD), 30 CN and 15 AD subjects were identified. In the fourth quadrant (refined: AD, conventional: CN), there were 11 CN and 12 AD subjects. Again, the refined EC method captured more true AD cases than the conventional method. Although only CN and AD subjects were included in this test visualization, *R*^2^ values were computed for all diagnostic groups (CN = 0.110, MCI = 0.325, AD = 0.117), indicating limited linear correspondence between the two segmentation methods across disease stages.

### 3.2 Diagnostic relevance of refined versus conventional EC segmentation

To evaluate the diagnostic relevance of different EC segmentation strategies, we performed comparative analyses using group-level statistics, feature importance metrics, and model interpretability tools. The two EC segmentation methods evaluated were the conventional EC, obtained from FreeSurfer, and the refined EC, derived from our nnU-Net–based model.

#### 3.2.1 Group-level discrimination

The refined EC volumes exhibited significantly greater group-level separability across clinical stages. One-way ANOVA revealed a strong main effect for the refined EC (*F* = 15.03, *p* = 2.48e−06), whereas the conventional EC did not yield statistically significant separation (*F* = 1.78, *p* = 0.175). These findings indicate that refined segmentation more effectively captures disease-related structural differences.

#### 3.2.2 Feature importance evaluation

**Figure 5:**
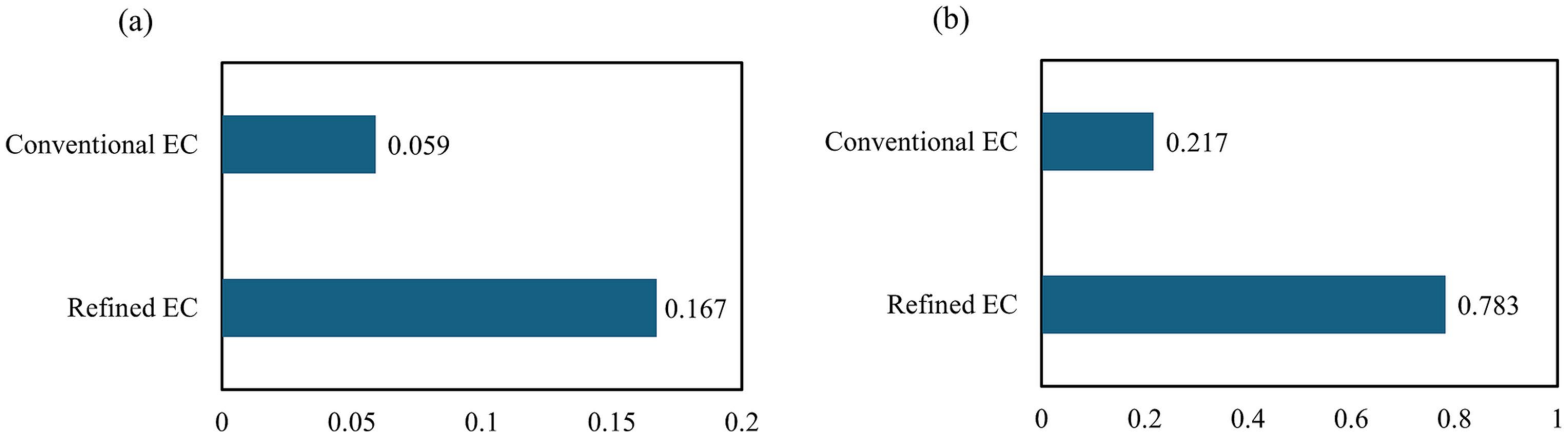
Feature importance scores for conventional and refined EC, derived from **(a)** permutation importance and **(b)** regression-based importance over 500 runs.

Feature importance analyses consistently favored the refined EC across both regression and classification tasks. In a regression framework modeling disease severity on a continuous scale, the refined EC volume achieved a markedly higher importance score (0.167) than the conventional EC (0.059). Similarly, in a classification framework, permutation-based importance was also higher for the refined EC (0.783 vs. 0.217). As illustrated in Figure 5, these results confirm the superior diagnostic informativeness of the refined segmentation.

#### 3.2.3 Model interpretability via SHAP analysis

**Figure 6:**
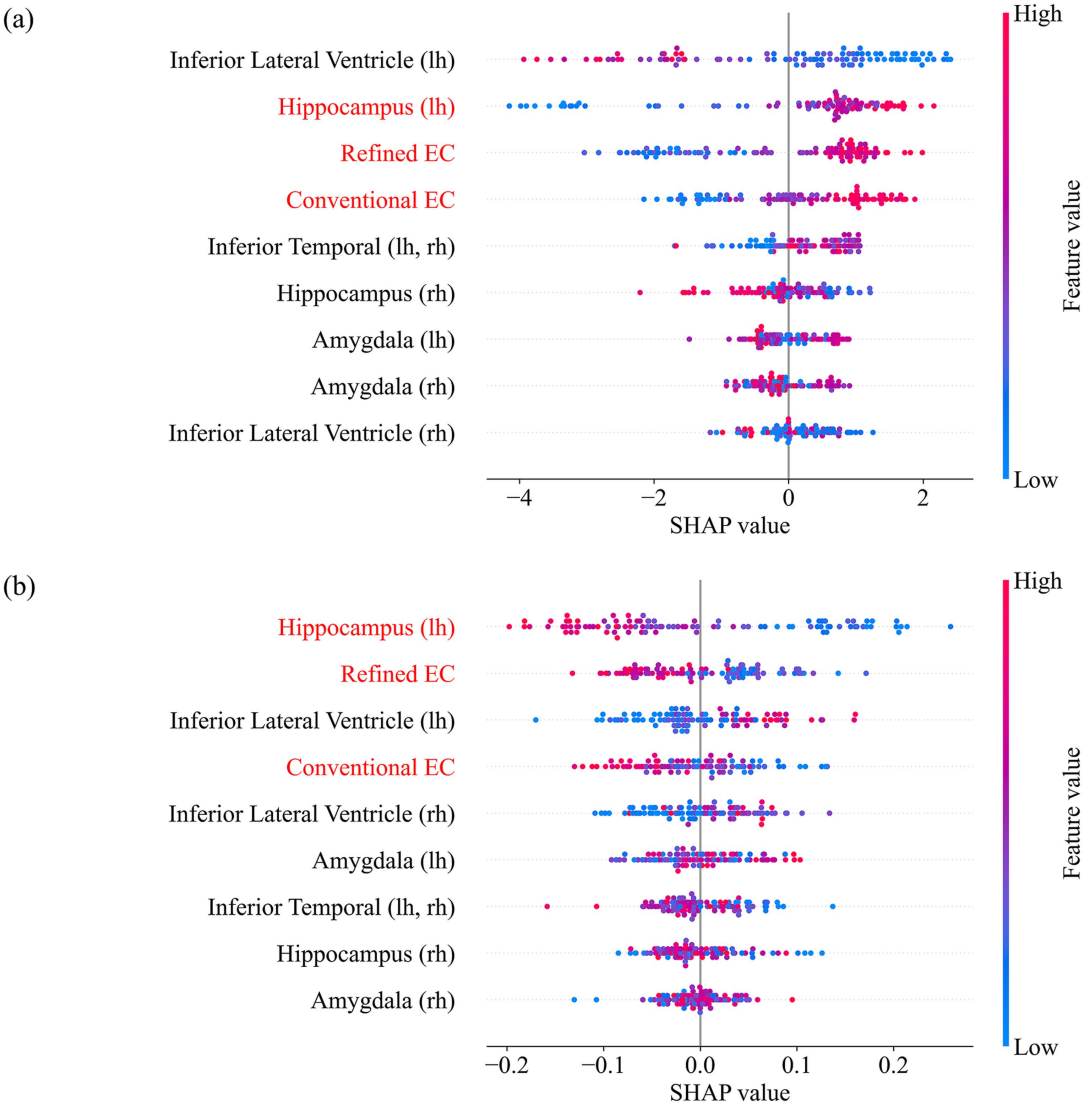
SHAP summary plots for models incorporating both EC variants. **(a)** Classification-based model distinguishing CN and AD. **(b)** Regression-based model predicting disease severity.

SHAP-based interpretability analysis further supported the refined EC’s clinical relevance. In regression models, the refined EC ranked second among all volumetric features in terms of mean SHAP value, while the conventional EC ranked fourth. In binary classification (CN vs. AD), the refined EC maintained a higher rank (third vs. fourth). Figure 6 presents the SHAP summary plots, highlighting the stronger and more consistent contribution of the refined EC to disease prediction models. These rankings demonstrate that refined EC volumes contribute more distinct and predictive information, even when included alongside other relevant anatomical features.

### 3.3 Classification results

As summarized in Table 3, compared with the conventional EC segmentation, the refined EC features generally improved classification performance across most tasks and classifiers.

**Table 3:** Classification performance on the ADNI1 dataset.

Task	Evaluation metric	Method	Model				
			RF	LR	SVM	XG	MLP
CN vs AD	F1 score	Conventional EC	0.883	0.888	0.900	0.840	0.900
		Refined EC	**0.896**	**0.911**	**0.921**	**0.874**	**0.930**
	AUROC	Conventional EC	0.922	0.919	0.924	0.919	0.924
		Refined EC	**0.941**	**0.953**	**0.955**	**0.922**	**0.966**
CN vs MCI	F1 score	Conventional EC	0.695	0.733	0.746	0.694	0.751
		Refined EC	**0.728**	**0.780**	**0.752**	**0.716**	**0.775**
	AUROC	Conventional EC	0.782	0.795	0.788	0.762	0.797
		Refined EC	**0.803**	**0.811**	**0.811**	**0.795**	**0.815**
MCI vs AD	F1 score	Conventional EC	0.692	**0.699**	0.690	0.668	0.692
		Refined EC	**0.724**	0.696	**0.690**	**0.685**	**0.721**
	AUROC	Conventional EC	0.687	0.691	0.626	0.664	0.692
		Refined EC	**0.713**	**0.734**	**0.741**	**0.681**	**0.759**

Classification performance (F1 score and AUROC) for three diagnostic tasks—CN vs. AD, CN vs. MCI, and MCI vs. AD—on the ADNI1 dataset. Five classifiers (RF, LR, SVM, XG, and MLP) were trained and evaluated using volumetric features extracted from either conventional EC segmentation (via FreeSurfer) or refined EC segmentation (generated by a deep learning model trained on expert-annotated labels). Refined EC features consistently yielded higher F1 scores and AUROC across most classifiers and tasks, demonstrating their superiority over conventional EC features in distinguishing clinical diagnostic groups. Bold values indicate the higher value between Conventional EC and Refined EC for each model–evaluation metric combination.

In the CN vs. AD task, performance gains were evident in both F1 and AUROC metrics. The MLP model achieved the highest F1 (0.930 vs. 0.900) and AUROC (0.966 vs. 0.924), corresponding to ~3–4% relative improvements. SVM (0.921 vs. 0.900) and LR (0.911 vs. 0.888) showed similar increases. Statistical testing confirmed that these improvements were significant (paired *t*-test *p* = 0.011–0.041; DeLong *p* = 0.007–0.020), indicating a more discriminative and balanced representation of disease-related morphometry.

In the CN vs. MCI classification, refined EC features again enhanced performance in most models, yielding moderate but consistent gains. LR (F1 = 0.780 vs. 0.733) and MLP (0.775 vs. 0.751) achieved the largest F1 improvements (≈ 3–5%), while AUROC increased from 0.795 to 0.811 on average. Several differences reached statistical significance (paired *t*-test *p* = 0.044–0.218; DeLong *p* = 0.026–0.061), suggesting that the refined EC captures subtle neuroanatomical alterations associated with early cognitive decline.

For the MCI vs. AD task, overall accuracy remained lower, reflecting the clinical overlap between these groups, yet refined EC features produced measurable gains in several classifiers. The MLP model achieved the highest F1 (0.721 vs. 0.692) and AUROC (0.759 vs. 0.692), representing ~4–5% relative gains, while others showed smaller or marginal changes. Statistical analysis confirmed significance for some models (paired *t*-test *p* = 0.057–0.904; DeLong *p* = 0.039–0.066).

Comprehensive pairwise comparisons and exact *p*-values are provided in Supplementary Table S1.

To assess generalizability, CN vs. AD classification was further evaluated using the independent MIRIAD dataset. As shown in Table 4, refined EC features again outperformed the conventional ones in most models. RF achieved the highest F1 (0.945 vs. 0.917), while MLP yielded the highest AUROC (0.992 vs. 0.976), corresponding to ~2–3% improvements. SVM also maintained strong generalization (F1 = 0.945 vs. 0.919; AUROC = 0.990 vs. 0.966). Statistical validation confirmed significant differences in multiple classifiers (paired *t*-test *p* = 0.061–0.392; DeLong *p* = 0.047–0.157), highlighting the robustness and external validity of the refined EC segmentation.

**Table 4:** Classification performance in MIRIAD dataset.

Task	Evaluation metric	Method	Model				
			RF	LR	SVM	XG	MLP
CN vs AD	F1 score	Conventional EC	0.917	0.899	0.919	0.894	0.922
		Refined EC	**0.945**	**0.939**	**0.945**	**0.914**	**0.926**
	AUROC	Conventional EC	0.979	0.956	0.966	0.957	0.976
		Refined EC	**0.988**	**0.987**	**0.990**	**0.981**	**0.992**

Classification performance (F1 score and AUROC) for the CN vs. AD task evaluated on the independent MIRIAD dataset. Five classifiers (RF, LR, SVM, XG, and MLP) trained on ADNI1 data were tested using features from conventional and refined EC segmentations. Refined EC features consistently outperformed conventional EC features across all classifiers, with RF achieving the highest F1 score (0.945) and MLP achieving the highest AUROC (0.992). These results demonstrate the strong generalizability of refined EC features to external clinical datasets. Bold values indicate the higher value between Conventional EC and Refined EC for each model–evaluation metric combination.

Detailed statistical comparisons are summarized in Supplementary Table S2.

## 4 Discussion

This study presents a refined EC segmentation framework that integrates expert-guided anatomical correction with a deep learning model. The primary motivation was to overcome persistent limitations in conventional MRI-based pipelines—most notably FreeSurfer, which inherits the DK atlas and often includes anterior perirhinal regions within the EC label. Such inclusion introduces structural and functional heterogeneity that can obscure disease-relevant atrophy patterns, thereby reducing the specificity of EC volume as a biomarker for AD. Given the EC’s pivotal role as one of the earliest cortical regions affected by AD, achieving anatomically faithful delineation is fundamental for improving the sensitivity of MRI-based diagnostic models.

Refining the EC boundary is particularly challenging due to its small size, irregular curvature, and proximity to the perirhinal cortex and collateral sulcus. These anatomical complexities often produce ambiguous boundaries even in high-resolution MRI, resulting in intersubject variability and inconsistent measurements across pipelines. To address these challenges, we developed a hybrid framework combining manual expert correction with the self-configuring nnU-Net. While the nnU-Net automatically optimizes network parameters based on input characteristics, expert supervision ensures conformity to histologically defined borders. This synergy between expert anatomical guidance and data-driven adaptation (Figure 1b) enables robust, reproducible segmentation for a structure that has long been difficult to delineate accurately.

Unlike general-purpose tools such as FastSurfer or TotalSegmentator, which focus on whole-brain or multi-organ parcellation, our framework targets cortical microanatomy and boundary fidelity. FastSurfer replicates FreeSurfer’s parcellation using deep learning but retains its anatomical inaccuracies, including the absence of clear separation between the entorhinal and perirhinal cortices. TotalSegmentator, while incorporating expert-verified labels, extends nnU-Net toward large-scale multi-organ segmentation primarily in CT and whole-brain MRI data. In contrast, our framework refines FreeSurfer-derived EC labels by selectively removing anterior perirhinal extensions and other anatomically inconsistent regions before retraining, representing an anatomy-informed, expert-in-the-loop paradigm. Manually corrected EC labels trained the initial network, neurologists reviewed model predictions, and the refined outputs were retrained to optimize microanatomical accuracy. This design effectively embeds expert neuroanatomical reasoning within a scalable deep learning process, advancing automated delineation of small, functionally specialized cortical regions. Conceptually, the proposed expert-in-the-loop framework aligns with hybrid optimization paradigms, where deterministic learning is iteratively refined through auxiliary supervision (Ali et al., 2025a) to achieve stable convergence and robustness. To assess its practical value, we compared the refined EC segmentation with conventional FreeSurfer-based delineation across diagnostic groups and examined how anatomical correction affected volumetric and diagnostic outcomes.

Figure 3a demonstrates that refined EC volumes are consistently smaller than those obtained from conventional segmentation across all diagnostic groups. However, this reduction is not a uniform downscaling. In CN subjects, refined EC volumes were reduced to 51.6% of the conventional measurement, whereas in MCI and AD groups the reductions were more pronounced—47.9 and 45.8%, respectively—corresponding to an additional 3.7 and 5.8% decrease with advancing disease severity. This progressive pattern indicates that the refined method selectively excludes anterior perirhinal regions that become increasingly incorporated within the conventional EC label as atrophy progresses. Consequently, the refined segmentation provides sharper anatomical differentiation in later disease stages. Notably, the relationship between conventional and refined EC volumes showed low linear correlation, particularly in MCI and AD, suggesting that the refined EC is not a compressed version but a structurally distinct delineation. The growing discrepancy with disease severity further underscores its potential to more accurately reflect AD-related EC atrophy.

Beyond volumetric comparison, the quadrant-based analysis of EC/ICV ratios (Figures 3b, 4) provided additional diagnostic insight. By jointly visualizing both segmentations and their classification concordance across quadrants, we observed that the refined EC often corrected misclassifications from the conventional approach, especially in AD cases. These distributions illustrate how improved anatomical precision enhances subject-level diagnostic validity. Although the advantage was somewhat reduced in nnU-Net–generated test predictions, the same structural trend persisted—highlighting that while deep learning ensures reproducibility, high-quality expert-defined labels remain critical for maintaining biological accuracy.

The refined model also maintained consistent geometric performance across disease stages (Table 2), indicating robustness against progressive cortical thinning and sulcal deformation. Slight declines in overlap metrics in the MCI group likely reflect transitional variability rather than segmentation failure, while lower surface-distance values in AD may partly result from reduced cortical surface area due to atrophy. Overall, these findings confirm that the framework achieves reliable anatomical definition even under pronounced structural changes.

At the feature level, the refined EC exhibited a significant main effect of diagnosis in one-way ANOVA (*p* < 0.001), whereas the conventional EC did not, demonstrating that anatomically faithful delineation more effectively captures disease-related variation among CN, MCI, and AD. Feature-importance analyses (Figure 5) further revealed that the refined EC contributed more strongly to model predictions across both regression- and permutation-based evaluations, confirming its superior diagnostic relevance within the multiregional feature ensemble. SHAP analyses (Figure 6) supported this finding: while hippocampal volume remained the dominant predictor—consistent with well-established neuropathology showing greater left hippocampal vulnerability in early AD—the refined EC consistently ranked above the conventional EC, indicating a stronger and anatomically specific contribution to diagnostic prediction.

At the model level, the performance results (Table 3) and statistical validation (Supplementary Table S1) consistently confirmed the benefit of incorporating the refined EC features. Pairwise statistical comparisons across five-fold cross-validation demonstrated significant improvements in F1-score for multiple classifiers (paired *t*-test *p* = 0.011–0.904) and corresponding AUROC gains (DeLong *p* = 0.007–0.066) across all diagnostic tasks. These findings indicate that the refined EC contributes meaningful and statistically reliable enhancement in classification performance. Importantly, even when the conventional EC was replaced with the refined EC while hippocampal features were kept identical, the models achieved higher diagnostic accuracy and better discriminative balance. This observation implies that the refined EC segmentation provides complementary, anatomically specific information beyond what is captured by neighboring hippocampal structures. The consistent performance improvements—most prominent in CN–AD and CN–MCI distinctions but smaller in MCI–AD—highlight that greater anatomical precision in EC delineation directly enhances sensitivity to subtle, stage-dependent neurodegenerative changes. Overall, these results demonstrate that anatomical fidelity within the EC region reinforces statistical robustness and diagnostic reliability, linking morphometric precision to meaningful clinical separability.

When applied to the independent MIRIAD dataset, the refined EC demonstrated strong generalizability. Models incorporating refined EC features consistently outperformed those using conventional segmentation across all classifiers, with significant gains in AUROC and F1 (Table 4). Interestingly, the model—trained solely on ADNI1 data—performed even better on MIRIAD, likely reflecting that dataset’s more homogeneous diagnostic criteria and standardized acquisition. Further validation on heterogeneous cohorts such as OASIS-3 (LaMontagne et al., 2019) and AIBL (Ellis et al., 2009) will be important to confirm robustness across scanner types, demographics, and imaging conditions. Given nnU-Net’s ability to adapt preprocessing and normalizing intensities, the framework is expected to remain stable under moderate heterogeneity. Future studies will extend these analyses to multi-center datasets and investigate domain adaptation strategies to harmonize EC segmentation across sites. In addition, integrating bio-inspired or fractional-order optimization algorithms (Ali et al., 2024; Ali et al., 2025a; Ali et al., 2025b) may further enhance segmentation generalizability under inter-scanner and inter-dataset variability.

While these results are promising, several limitations should be acknowledged. Manual expert correction, though essential for anatomical precision, remains time-consuming and may limit scalability. Future directions include integrating self-supervised (Jing and Tian, 2020) or generative (Huo et al., 2025) learning approaches to reduce annotation burden while maintaining accuracy. Recent advances in contrastive representation learning (Wang et al., 2023) and diffusion-based segmentation (Wu et al., 2024) could enable automated refinement using unlabeled data, potentially achieving expert-level delineation at scale. Second, as this work focused on cross-sectional data, longitudinal analyses are warranted to determine whether refined EC metrics serve as stable biomarkers for disease progression and therapeutic monitoring. Finally, the standard 1 mm^3^ resolution of T1-weighted MRI limits precise delineation of thin cortical structures such as the EC, suggesting that super-resolution (Chen et al., 2018) or diffusion-based reconstruction (Webber and Reader, 2024) could further enhance morphological detail.

Despite these limitations, the refined EC segmentation provides both clinical and methodological value. Its anatomically grounded delineation offers a reproducible biomarker for early AD detection, improving diagnostic sensitivity and supporting accurate disease staging. Clinically, it may facilitate patient stratification in therapeutic trials targeting prodromal or early disease phases, where subtle cortical degeneration is most relevant. From a methodological perspective, the proposed expert-in-the-loop design bridges neuroanatomical expertise with scalable automation, providing a transferable blueprint for refining other complex cortical regions such as the perirhinal, fusiform, or insular cortices.

In summary, the refined EC segmentation improves group differentiation, enhances model interpretability, and strengthens generalization across datasets, suggesting a promising direction for anatomically informed deep learning in neuroimaging-based diagnosis.

## Data Availability

Publicly available datasets were analyzed in this study. This data can be found at: http://adni.loni.usc.edu.
