# Supplementary material for: Anatomically refined entorhinal cortex segmentation improves MRI-based early diagnosis of Alzheimer’s disease
Source: Front Aging Neurosci. 2025 Dec 3;17:1682106. doi: 10.3389/fnagi.2025.1682106 (PMC12708528; doi:10.3389/fnagi.2025.1682106)
Supplement: Supplementary file 1 [file Table_1.DOCX]

**Table S1.** Statistical comparison of model performance on the ADNI1 dataset across diagnostic tasks.

| **Task** | **Evaluation Metric** |  |  | **Model** |  |  |
| --- | --- | --- | --- | --- | --- | --- |
|  |  | **RF** | **LR** | **SVM** | **XG** | **MLP** |
| **CN vs AD** | **t-test (F1)** | **0.034** | **0.021** | **0.018** | **0.041** | **0.011** |
|  | **DeLong (AUROC)** | **0.012** | **0.010** | **0.008** | **0.020** | **0.007** |
| **CN vs MCI** | **t-test (F1)** | 0.068 | 0.052 | 0.218 | 0.083 | **0.044** |
|  | **DeLong (AUROC)** | **0.038** | **0.029** | 0.061 | **0.047** | **0.026** |
| **MCI vs AD** | **t-test (F1)** | 0.094 | 0.612 | 0.904 | 0.182 | 0.057 |
|  | **DeLong (AUROC)** | 0.059 | **0.048** | 0.051 | 0.066 | **0.039** |

Pairwise statistical comparisons were performed to assess performance differences across five cross-validation folds on the ADNI1 dataset. Paired *t*-tests were applied to F1-scores to evaluate fold-wise differences, and DeLong tests were conducted on AUROC values computed from aggregated OOF prediction scores. Bold values indicate statistically significant differences (*p* < 0.05).

**Table S2.** Statistical comparison of model performance on the external MIRIAD dataset.

| **Task** | **Evaluation Metric** |  |  | **Model** |  |  |
| --- | --- | --- | --- | --- | --- | --- |
|  |  | **RF** | **LR** | **SVM** | **XG** | **MLP** |
| **CN vs AD** | **t-test (F1)** | 0.061 | 0.083 | 0.072 | 0.118 | 0.392 |
|  | **DeLong (AUROC)** | **0.047** | 0.064 | 0.052 | 0.089 | 0.157 |

Pairwise statistical comparisons were performed to evaluate generalization performance across models using the external MIRIAD dataset. The paired *t*-test assessed fold-wise differences in F1-scores, and the DeLong test compared AUROC distributions computed from the OOF prediction scores aggregated across all folds. Bold values indicate statistically significant differences (p < 0.05).
